# Biomechanical assessment of anterior plate system, bilateral pedicle screw and transdiscal screw system for high-grade spondylolisthesis: a finite element study

**DOI:** 10.3389/fbioe.2024.1491420

**Published:** 2024-11-28

**Authors:** Utpal K. Dhar, Hadi Sultan, Kamran Aghayev, Chi-Tay Tsai, Frank D. Vrionis

**Affiliations:** ^1^ Department of Ocean and Mechanical Engineering, Florida Atlantic University, Boca Raton, FL, United States; ^2^ Charles E. Schmidt College of Medicine, Florida Atlantic University, Boca Raton, FL, United States; ^3^ BHT Clinic, Istanbul, Türkiye; ^4^ Department of Neurosurgery, Marcus Neuroscience Institute, Boca Raton Regional Hospital, Boca Raton, FL, United States

**Keywords:** spondylolisthesis, finite element study, internal fixation technique, range of motion, interbody cage

## Abstract

**Introduction:**

Limited information regarding the biomechanical evaluation of various internal fixation techniques for high-grade L5-S1 spondylolisthesis is available. The stiffness of the operated segment and stress on the hardware can profoundly influence clinical outcomes and patient satisfaction. The objective of this study was to quantitatively investigate biomechanical profiles of various fusion methods used for high-grade spondylolisthesis by using finite element (FE) analysis.

**Methods:**

An FE lumbar spine model of healthy spine was developed based on a patient’s CT scan. High-grade (III-IV) spondylolisthesis (SP model) was created by sliding L5 anteriorly and modifying L5-S1 facet joints. Three treatment scenarios were created by adding various implants to the model. These scenarios included L5-S1 interbody cage in combination with three different fixation methods–the anterior plate system (APS), bilateral pedicle screw system (BPSS), and transdiscal screw system (TSS). Range of motion (ROM), von Mises stress on cage, internal fixation as well as on the adjacent annuli were obtained and compared. The resistance to slippage was investigated by applying shear force on L5 vertebra and measuring its displacement regarding to S1.

**Results:**

Under different loading conditions all treatment scenarios showed substantial reduction of ROM in comparison with SP model. No notable differences in ROM were observed between treatment models. There was no notable difference in cage stress among models. The von Mises stress on the internal fixation in the TSS model was less than in APS and BPSS. The TSS model demonstrated superior resistance to shear load compared to APS and BPSS. No discernible difference was observed between the SP, APS, BPSS, and TSS models when compared the ROM for adjacent level L4-L5. TSS’s von Mises stress of the adjacent annulus was higher than in APS and BPSS.

**Conclusions:**

The TSS model exhibited biomechanical superiority over the APS and BPSS models.

## Introduction

Lumbar spondylolisthesis is a significant contributor to lower back pain and disability worldwide (Hussain et al., 2019; Sevrain et al., 2012; Natarajan et al., 2003; Kerr et al., 2021). Spondylolisthesis is defined as anterior translation of upper vertebra relative to the lower one and frequently occurs at the L5-S1 level. It has been categorized into six types such as pathologic, dysplastic, isthmic, traumatic, postsurgical, and degenerative (Bydon et al., 2019). Spondylolistheses is often classified in four grades, grade I is a slippage of 0%–25%, grade II is a slippage of 25%–50%, grade III is a slippage of 50%–75%, and grade IV is a slippage of 75%.

High-grade spondylolisthesis is defined as slippage degree by more than 50% and constitutes to 11.3% of all spondylolisthesis cases (Kalichman et al., 2009; Beck et al., 2019). Patients may be asymptomatic in the initial stages and slowly progress to grade III-IV. Spondylolisthesis can have a substantial effect on patients and is known to be a significant driver of morbidity (Suzuki and Nakamura, 2022; Yang et al., 2021; Hermansen et al., 2022). The lumbosacral junctional area has the highest incidence of spondylolisthesis due to the higher motion and dynamic load.

Although, low-grade spondylolisthesis could be treated with physical therapy to prevent further slippage, high-grade cases usually require surgical intervention (Kerr et al., 2021; Kunze et al., 2020; Natarajan et al., 2003). Surgery includes decompression and stabilization which is accomplished by removing bone and ligaments as well as fixing the two vertebrae together. Such stabilization is best achieved by interbody fusion technique. This procedure comprises removal of the disc material (discectomy) and insertion of interbody cage and bone graft into the discectomy cavity. Although the cage has excellent vertical load-bearing capacity it cannot stabilize the segment. Thus, the stability is restored and enhanced by pedicle screw-rod system or anterior plate-screw system (Ye et al., 2022; Chen et al., 2021). Such a combination of interbody cage/graft with either anterior or posterior fixation is the strongest stabilization method and yields the best clinical outcomes. Globally, companies are striving to create commercially viable implants for spinal fusion, aiming to achieve aligned bony fusion (Ohta et al., 2011). The stabilized segment must develop fusion, i.e., bone bridging for long-term benefit. If fusion fails to develop the condition is known as nonunion, pseudofusion or pseudoarthrosis. Most of the cases of pseudoarthrosis necessitate revision surgery (Chan et al., 2019; Minamide et al., 2019). Nonunion may result in breakage or loosening of the hardware. Incorporating internal fixation lowers the risk of nonunion in posterolateral fusion (Guigui and Ferrero, 2017). Some studies suggest that the anterior fusion has many advantages over the posterior fusion as the surface area is greater (Beck et al., 2019; Molinari et al., 2002). Hence, in comparison to posterior constructs, anterior support showed a reduced rate of pseudarthrosis. In any case, nonunion usually entails pain and radiculopathy and is very difficult to treat (Tumialán, 2019). Successful fusion relies on the strength of stabilization. In other terms, the higher the stiffness of the stabilized segment the higher the chance of fusion and successful clinical outcome (Chen et al., 2021; Cho et al., 2017). Recently a novel stabilization technique was developed and reported for L5-S1 level (Aghayev et al., 2024). This method includes interbody cage/graft insertion in combination with transdiscal screws. The screws are inserted from sacrum directly to L5 vertebral body through the disc space. The first transdiscal screw fixation for high grade L5-S1 spondylolisthesis was reported by Abdu et al. (Abdu et al., 1994) and included S1-L5 transdiscal, L4 pedicle screws connected with rods. The authors concluded that the method was safe and effective, yet they immobilized the L4-L5 segment, which was unnecessary. This idea was followed by others and several modifications were reported (Kerr et al., 2021; Lakshmanan et al., 2009). For example, Lakshmanan et al. (Lakshmanan et al., 2009), used transdiscal hollow screws in combination with standard pedicle screws and achieved good circumferential fusion. The placement of transdiscal screw is not technically challenging in high-grade L5-S1 spondylolisthesis, and the use of transdiscal screws is promising as it offers comparable or better rigidity to pedicle screw fixation while providing the advantage of reducing the surgical time (Chen et al., 2021).

FE analysis is an alternative biomechanical evaluation method and has several advantages that difficult to be obtained from cadaveric studies such as measuring stress on spinal structures and hardware (Natarajan et al., 2003; Sevrain et al., 2012; Zhu et al., 2017; Liu C. et al., 2024). FE analysis has been used to investigate both low- and high-grade spondylolisthesis for different surgical levels of the lumbar spine (Zhu et al., 2017; Ye et al., 2022; Liu C. et al., 2024; Lv et al., 2018). To authors’ knowledge, this study is the first to analyze both anterior and posterior fixation techniques for high-grade spondylolisthesis. Additionally, a novel transdiscal screw system has been evaluated and compared to standard techniques. Previously a study included FE analysis of TSS (Aghayev et al., 2024) in low grade spondylolisthesis and non-spondylolisthesis cases. However, this study specifically investigated high grade spondylolisthesis model.

Research on the biomechanical performance of various internal fixation techniques for high-grade L5-S1 spondylolisthesis is limited. The stability of the treated segment and the forces on the hardware may strongly influence clinical success and patient contentment. The purpose of this study was to quantitively analyze the ROM and stress distribution in the adjacent annulus, cage, and screw for different internal fixation techniques for the high-grade spondylolisthesis.

## Methods and materials

### Intact spondylolisthesis model (SP model)

**FIGURE 1:**
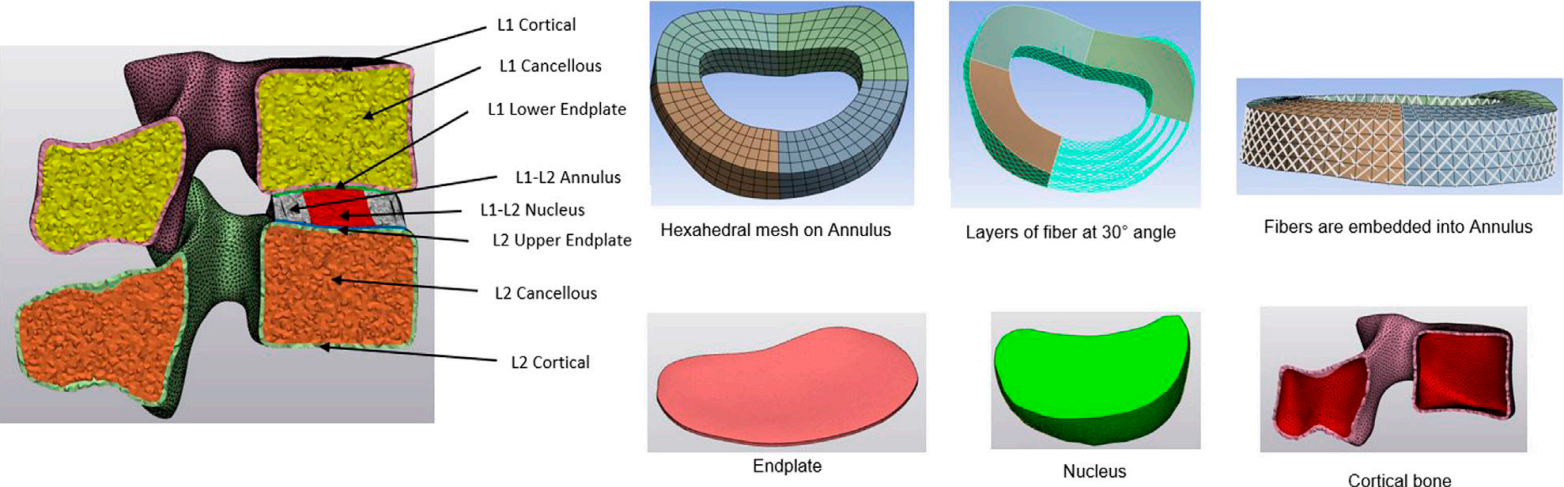
Detailed view of the L1-L2 segment, parts of the intervertebral disc, and vertebra.

Using CT imaging of a healthy patient (aged 64, Female), a comprehensive 3D non-linear lumbar FE model encompassing the entire L1-Sacrum segment was developed. Initially, the DICOM data was transferred into Mimics 25.0 software (Materialize, Leuven, Belgium), where the vertebral body segments were isolated through image threshold segmentation alongside processes such as removing, filling, smoothing, wrapping, and other related functions. Then, the vertebral body was imported to 3-Matic software to create the cortical and cancellous bone, as well as the annulus, nucleus, and endplate. The thickness of the cortical bone was set to 1 mm, and the endplate was 0.5 mm (Edwards et al., 2001). After creating the intervertebral disc, the annulus and nucleus were created for different levels. The cortical and cancellous bone, endplate, and nucleus were chosen as tetrahedral meshes, and the annulus mesh was hexahedral to add the annulus fibers (Wang and Guo, 2022). The annulus fibrosus was presumed to be a composite material comprising of fibers embedded within a uniform matrix material, and eight layers of fibers were added, alternating at an inclination approximately at a 30° angle to the horizontal plane (Schmidt et al., 2007). The Young modulus increases linearly from the innermost fibers (360 MPa) to the outermost fibers (550 MPa) (Shin et al., 2007; Eberlein et al., 2004; Bereczki et al., 2021; Wang and Guo, 2022). A detailed view of the intervertebral disc and vertebral components for the L1-L2 is shown in Figure 1. The intervertebral disc functions as a poroelastic material comprising a solid matrix and fluid-filled pores that respond to applied loads. Serpieri et al.(Serpieri and Travascio, 2016) derived a general operative formula that can be applied to partial phase stresses in response to externally applied stress as a function of partitioning coefficients.

Finally, the meshed FE model was loaded into Ansys Workbench (Ansys Workbench 2023 R1, Ansys Inc. Canonsburg, PA, United States), and seven types of major ligaments such as posterior and anterior longitudinal, facet capsular, flavum, intertransverse, interspinous, supraspinal ligaments were attached. Ligaments and annulus fibers were replicated using tension-only spring and bar elements, respectively. Anterior spondylolisthesis was simulated by anteriorly shifting L5 over S1 while keeping the S1 vertebra immobile. The facet of the L5 was elongated and oriented transversely instead of vertically. Finally, the grade III-IV spondylolisthesis was created between L5-S1.

**FIGURE 2:**
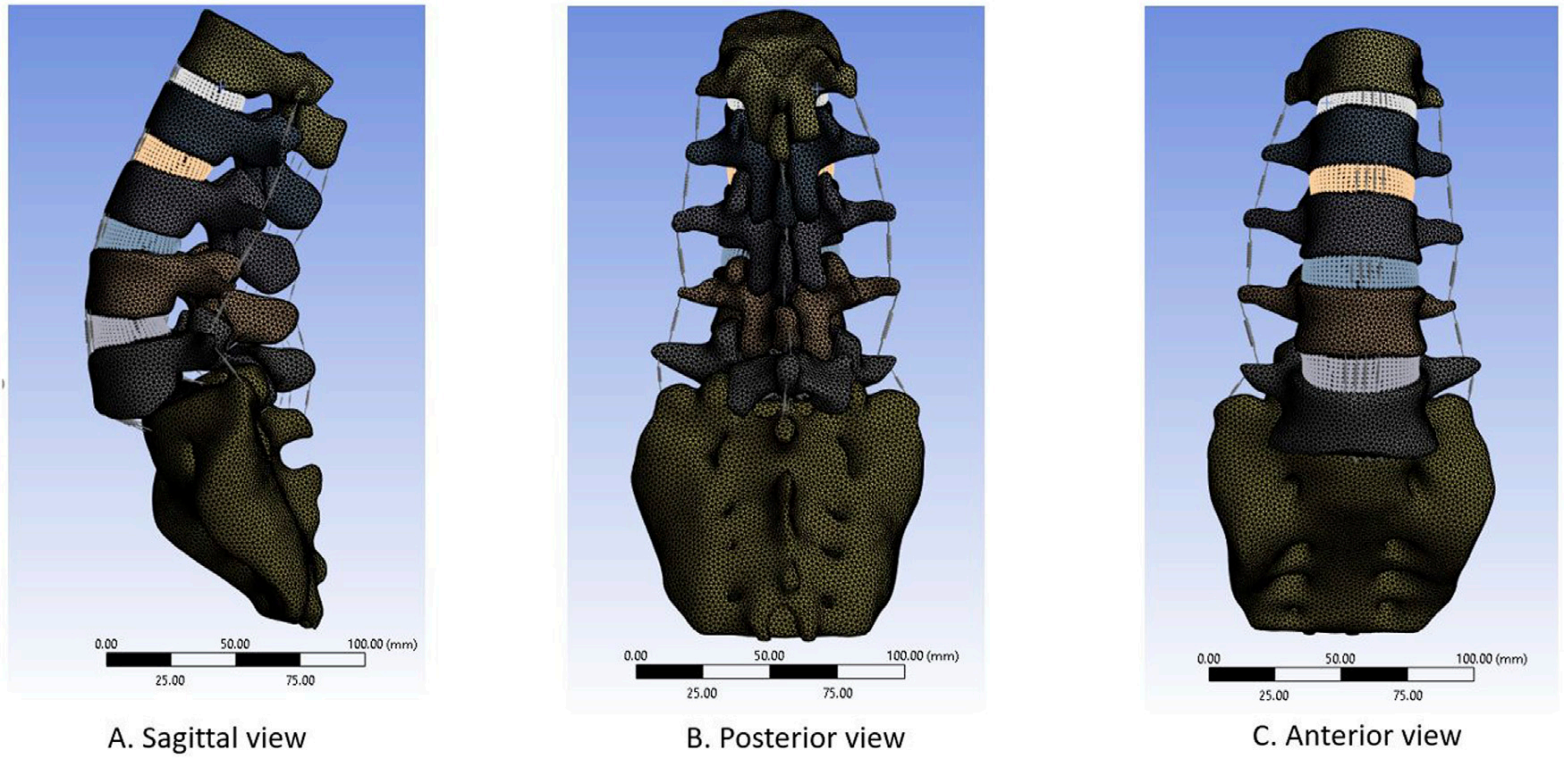
Complete FE model for Spondylolisthesis L1-Sacrum. **(A)** Sagittal view **(B)** Posterior view. **(C)** Anterior view.

The lateral, posterior, and anterior views of spondylolisthesis model are depicted in Figure 2. The complete L1-Sacrum model was comprised of 524,183 elements, 887,908 nodes, and material characteristics derived from previously documented literature (refer to Table 1). Non-linear force-deflection curves represented the response of each ligament to various physiological loading conditions were defined from prior experiments as per previous research findings (Zhang et al., 2022; Shirazi-Adl et al., 1984; Wang and Guo, 2022; Lv et al., 2018).

**TABLE 1:** Element type and material properties used in the model.

Component name (Ref.)	Number of element	Number of nodes	Young modulus (Mpa)	Poisson ratio
Cortical (Shirazi-Adl et al., 1984)	31,428	96,240	12,000	0.3
Cancellous (Shirazi-Adl et al., 1984)	52,695	279,045	100	0.2
Endplate (Panjabi et al., 2001)	195,583	318,275	500	0.45
Annulus ground (Shirazi-Adl et al., 1984)	4,480	7,500	4.2	0.45
Annulus fiber (Bereczki et al., 2021; Eberlein et al., 2004; Shin et al., 2007; Wang and Guo, 2022)	29,225	39,725	360–450	Cross-sectional area(0.15 mm^2^)
Ligament (Lv et al., 2018; Shirazi-Adl et al., 1984; Wang and Guo, 2022; Zhang et al., 2022)	Spring	Calibrated force-deflection curve		
Nucleus (Zhang et al., 2022)	210,772	147,123	1	0.4
Titanium (Lv et al., 2018)			110,000	0.3

### Model for anterior plate system (APS model)

**FIGURE 3:**
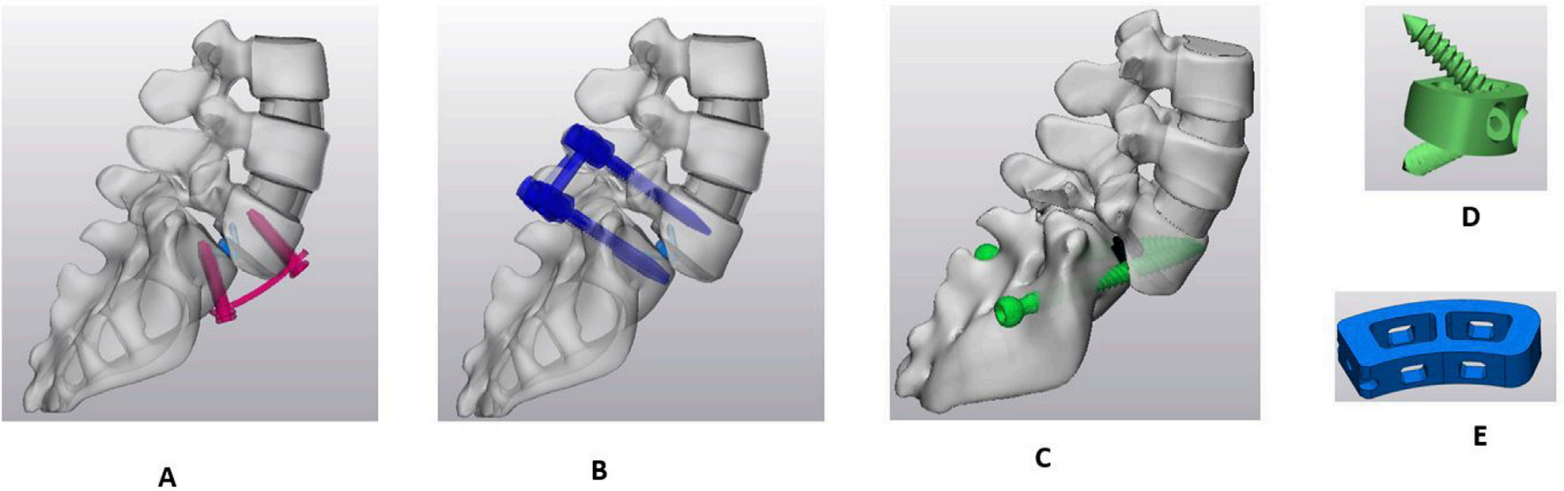
Sagittal view of Sacro-lumbar interbody fusion (SLIF) with three different fixation techniques. **(A)** Anterior plate system (APS). **(B)** Bilateral pedicle screw system (BPSS). **(C)** Transdiscal screw system (TSS). **(D)** ALIF cage. **(E)** TLIF cage.

The anterior longitudinal ligaments and nucleus were removed between L5 and sacrum. The anterior and posterior part of the annulus was also removed to insert the interbody cage. Following the partial extraction of the intervertebral disc, the dimension of the predetermined position of the interbody cage was confirmed to fit between L5-sacrum. Subsequently, the trapezoidal ALIF cage was crafted using CAD software (Solidworks 2023; Dassault Systèmes Inc., France), utilizing the measurements between the L5-sacrum, and was inserted. Two internal screws were incorporated with the ALIF cage and fixed it to L5 and S1 endplates. An anterior plate with four screws was placed at L5-S1. Figure 3A shows the APS model with the ALIF cage with the anterior plate and four screws.

### Model for bilateral pedicle screw system (BPSS model)

The anterior longitudinal ligament was removed the same as in the APS model, and the exact shape of a trapezoidal cage was inserted between the L5-sacrum. The pedicle screws and rods were designed in Solidworks 2023 and inserted to the L5 and S1 (Figure 3B).

### Model for transdiscal screw system (TSS model)

**FIGURE 4:**
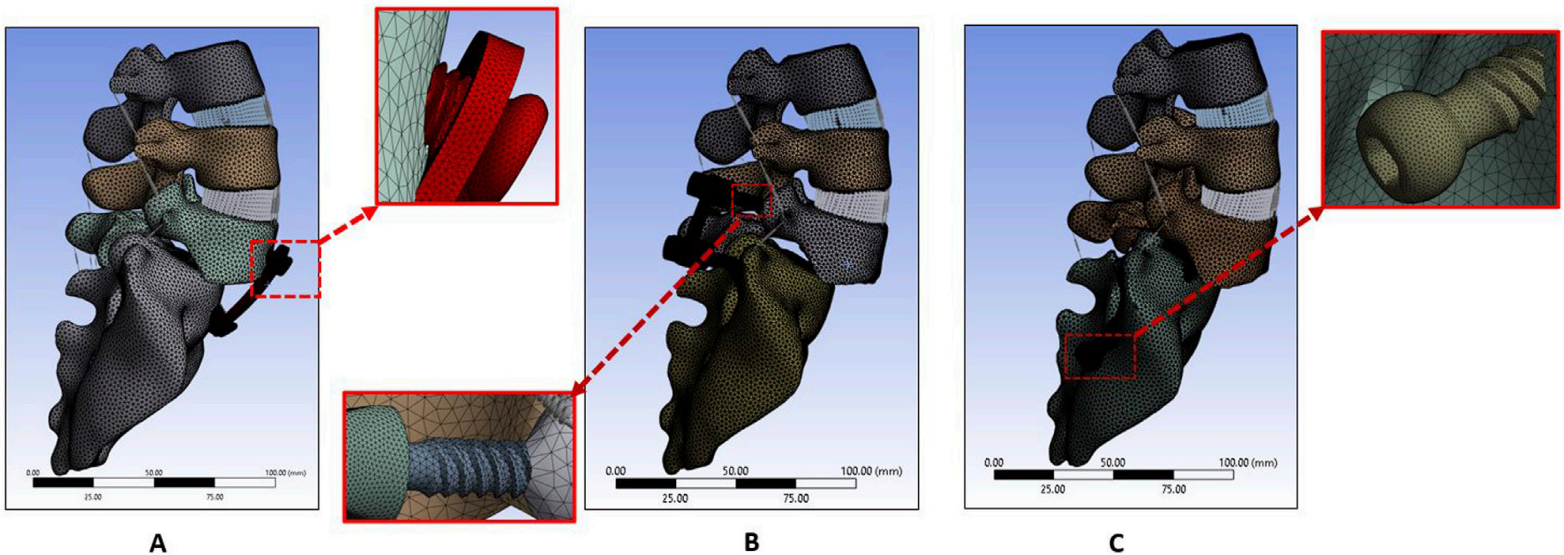
FE model for **(A)** Anterior plate system (APS model). **(B)** Bilateral pedicle screw system (BPSS model). **(C)** Transdiscal screw system (TSS model).

A unilateral facetectomy was performed on the L5-S1 (Figure 3C). The lateral parts of the flavum and posterior longitudinal ligaments were removed and a TLIF cage was inserted between L5-S1. Two transdiscal screws were inserted from the sacrum to L5 vertebra. The dimensions and route of the transdiscal screws was followed by previous publication (Aghayev et al., 2024). Detailed views of the FE model for the APS, BPSS, and TSS are illustrated in Figure 4. The material properties of pedicle screws, rod, anterior plate, transdiscal screws, and interbody cages were set to titanium.

### Contact, loading and boundary condition

The cage, screw, and vertebral body was set as a bonded connection to simulate the solid fusion. The contact between the screw tulip, nuts and rod was also set as bonded. The connection between the anterior plate and screw set as a bonded connection.

The sacrum was fixed in all directions, and a 7.5 Nm moment with a 500N compressive force was applied to the upper endplate of the top vertebrae to simulate flexion-extension, lateral bending, and axial rotation. Regarding shear load, a compressive force of 500N, together with a shear load of magnitude 150N, was applied at the center of the L5 vertebra anteriorly in a direction parallel to S1 endplate. After applying the compressive and shear load, the L5 vertebral displacement was measured while the sacrum was fixed in all directions. The displacement due to anterior shear loading for APS, BPSS and TSS models was compared. These loads replicated the loading scenarios noticed in the mechanical assessment of the cadaveric motion segments (Nachemson et al., 1979; Panjabi et al., 1984).

## Results

### FE model validation

**FIGURE 5:**
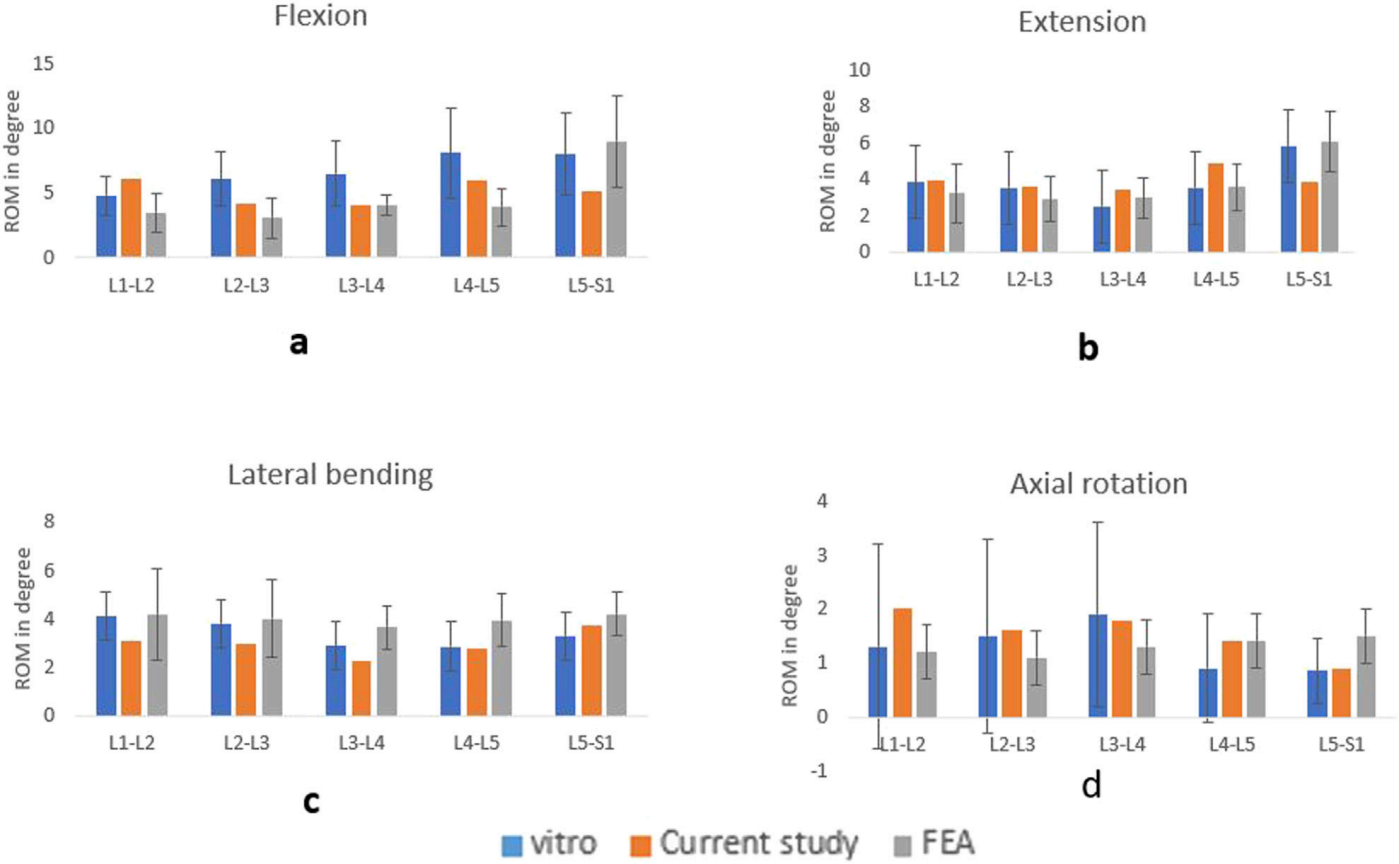
Comparison of ROM with the vitro and FEA study for flexion **(A)**, extension **(B)**, lateral bending **(C)**, axial rotation **(D)**.

Prior to the model validation, mesh convergence was performed. For the endplate and nucleus pulposus, a relatively fine mesh was chosen. Convergence assessments were conducted on the FE model to validate the mesh quality. For the endplate and nucleus pulposus, a relatively fine mesh was chosen. The mesh was deemed to converge when the variance between the predicted von Mises stresses of various components from two consecutive mesh refinements was below 5% under a moment of 7.5 Nm. The sacrum was constrained in all directions, and a 7.5 Nm moment along with a 500 N compressive load was applied to the superior endplate of the L1 vertebra to simulate flexion-extension, lateral bending, and axial rotation. The ROM was compared with those derived from a cadaveric investigation conducted previously *in vitro* experiments and other FEA studies. Figure 5 illustrates the ROM of the current study compared with the experimental and FEA results. The results showed congruence with the previously documented data (Panjabi et al., 1994; Dreischarf et al., 2014; Liu R. et al., 2024; Renner et al., 2007).

### ROM

#### L5-S1 ROM

**FIGURE 6:**
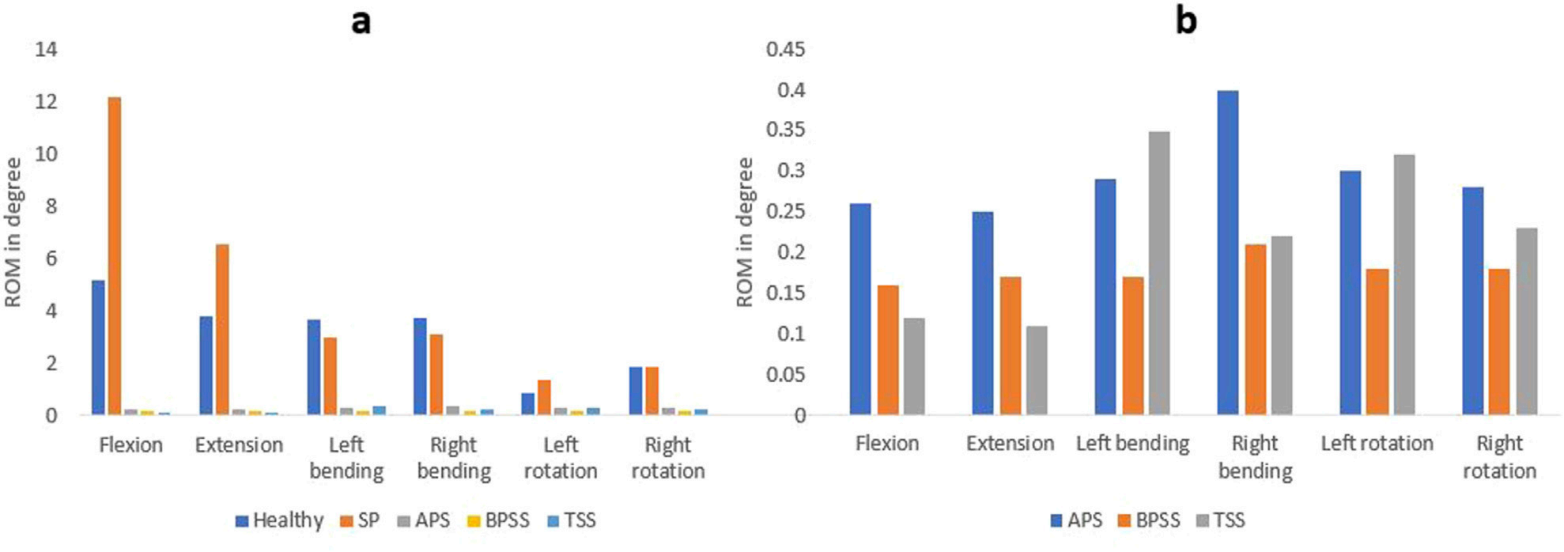
Comparing the ROM at the fusion level. **(A)** Between the healthy, SP, and surgical FE models. **(B)** Between the different surgical FE models.

Figure 6 shows the ROM for healthy, SP, APS, BPSS, and TSS models under six physiological loading conditions. The ROM experienced a notable decrease at the stabilized level for the APS, BPSS, and TSS models compared to the SP or healthy models. The FE analysis of the L5-S1 region in the SP model revealed ROM that closely resembled the biomechanical findings observed in the vitro model (Panjabi et al., 1994), except for the flexion condition. The highest ROM (12.16°) was observed in the SP model for the flexion situation, whereas 6.53° for the extension, 0.90° for left rotation and 3.71° for left bending. The ROM of the fused level decreases to 85%–95% for all loading scenarios for APS, BPSS, and TSS models. The TSS model showed the minimum ROM in L5-S1 when compared with APS or BPSS models. But the difference was negligible.

### L4-L5 ROM

**FIGURE 7:**
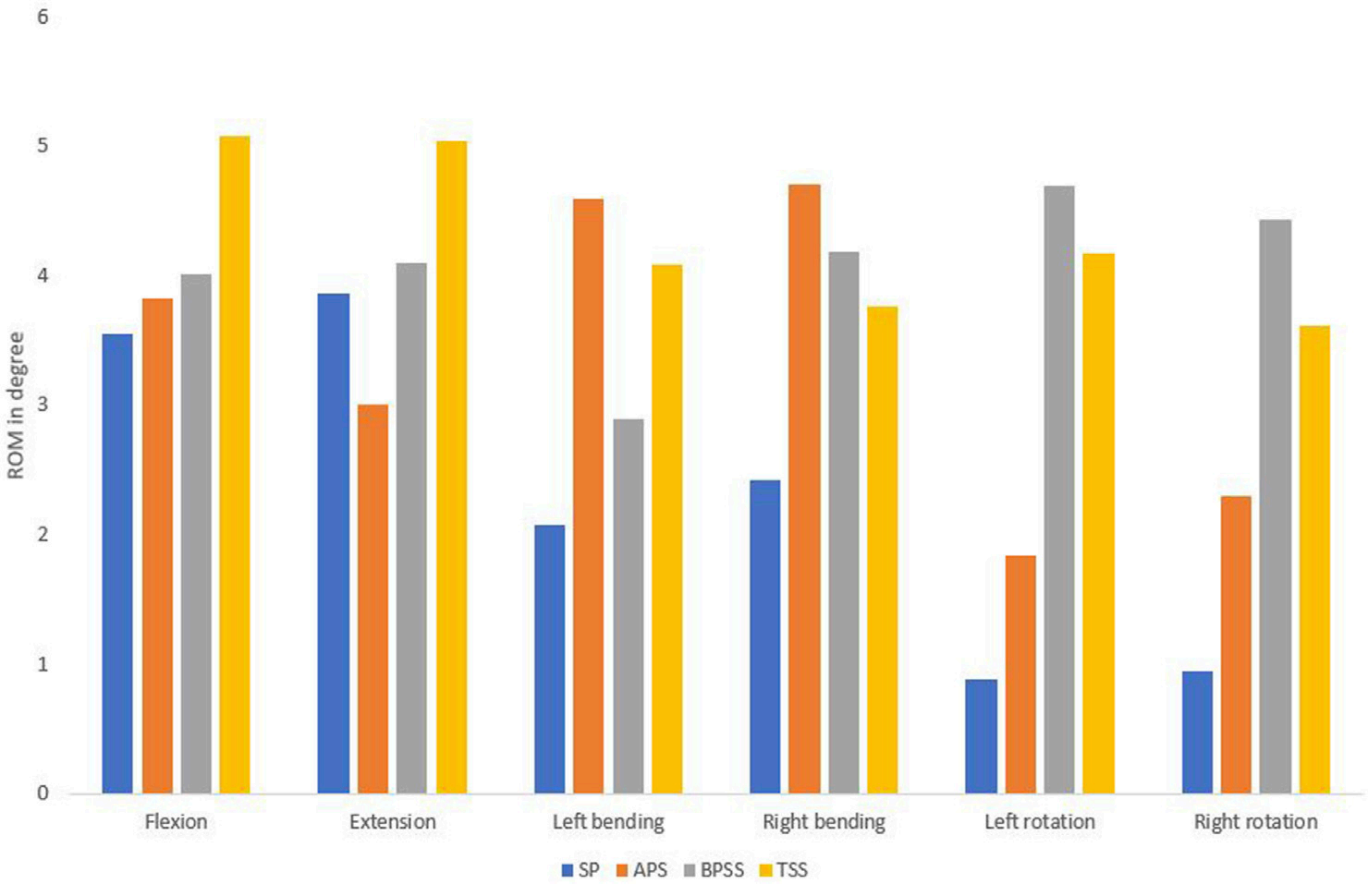
Comparing the range of motion between healthy, SP, APS, BPSS, and TSS models for flexion, extension, left axial rotation, right axial rotation, left bending, and right bending in the adjacent level for L4-L5.

The ROM for SP and treatment models was shown in Figure 7. The ROM of the APS, BPSS, and TSS models increases with respect to SP model for all loading conditions. The ROM of the TSS model exhibited approximately 30% higher than the APS and BPSS model for the flexion and extension. In left or right rotation, TSS and BPSS models showed four-fold higher than the SP model. No notable differences were noticed among the fused models for the lateral bending scenario.

### Stresses on the adjacent level

**FIGURE 8:**
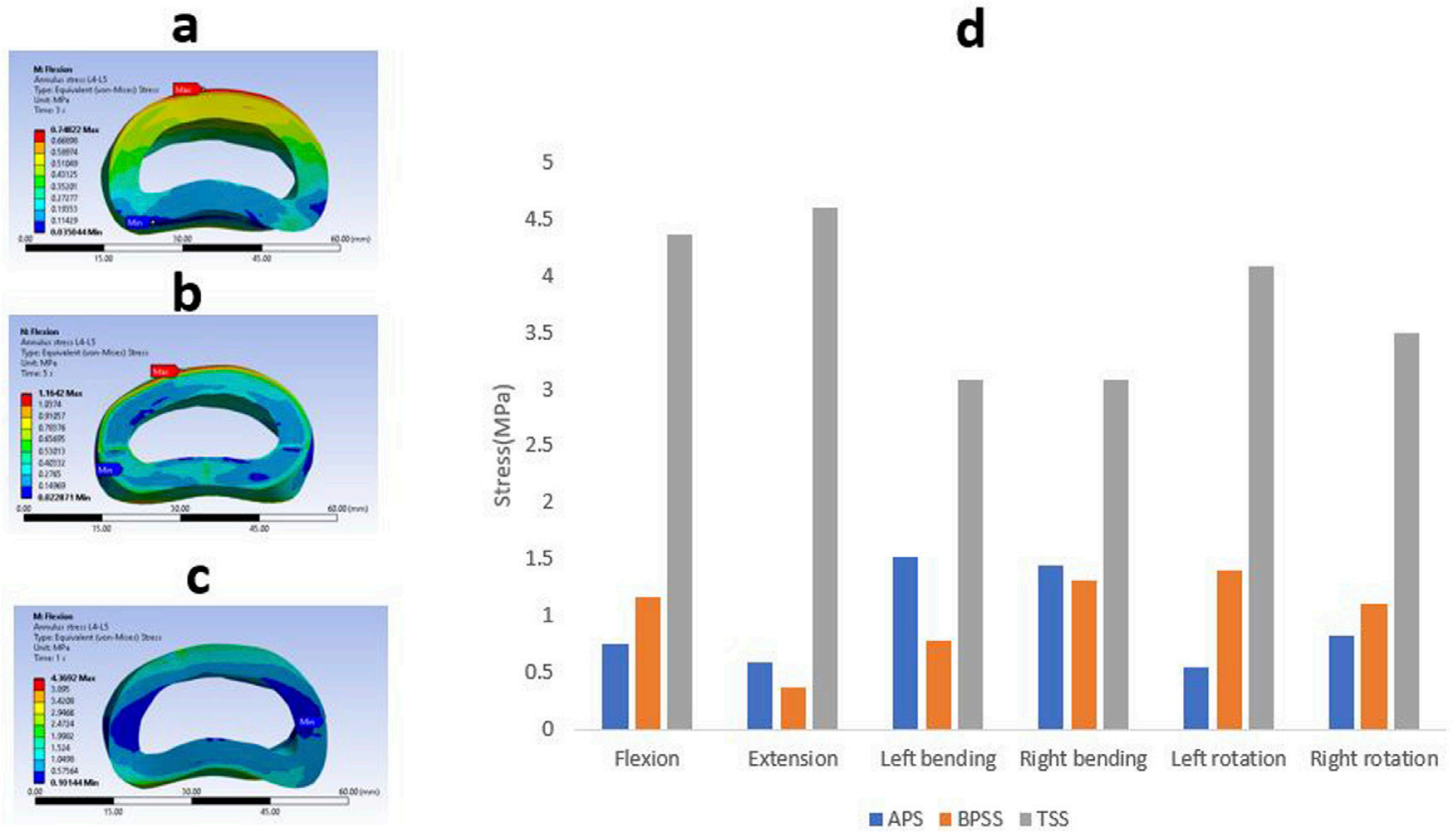
A typical von Mises stress contour for L4-L5 annulus under flexion condition **(A)** APS **(B)** BPSS **(C)** TSS model; **(D)** Comparison of von Mises stresses on adjacent annulus L4-L5 for flexion, extension, left axial rotation, right axial rotation, left bending, and right bending in different fixation techniques.

The Von Mises stresses on the adjacent annulus for APS, BPSS, and TSS are shown in Figure 8. No notable differences were noticed between the APS and BPSS for L4-L5 annulus. Of note, L4-L5 stress in TSS model showed more than 4-fold increase in comparison with BPSS and APS models for the flexion, extension and axial rotation. The lateral bending of the TSS model demonstrates over twice the stress when compared to the APS or BPSS model. Overall, the average stress within the annulus in the TSS model was higher than in the APS and BPSS models for all biomechanical loading conditions.

### Cage stress

**FIGURE 9:**
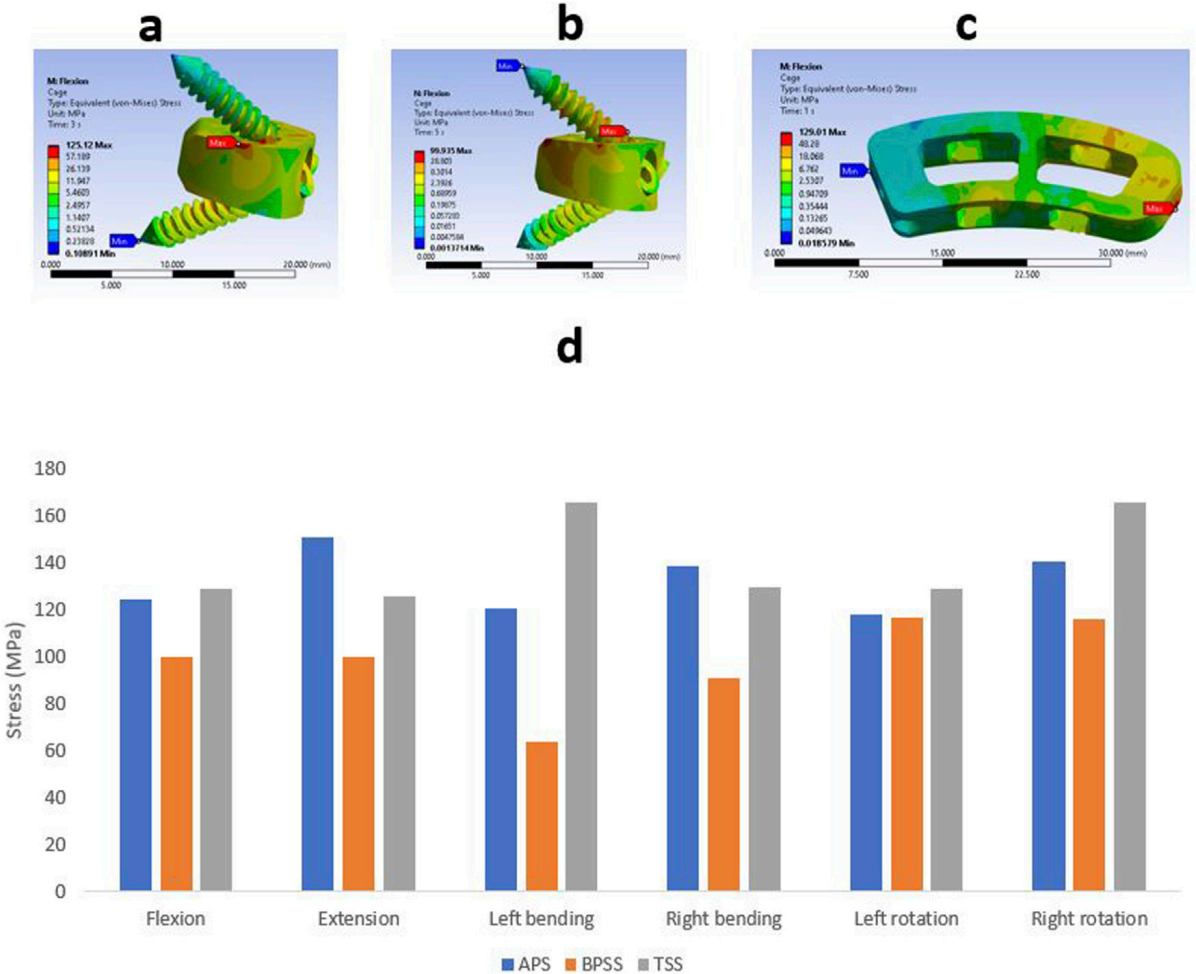
Von Mises stress contour on cage under flexion conditions **(A)** APS **(B)** BPSS **(C)** TSS model; **(D)** Comparison of von Mises stress on cage for APS, BPSS, and TSS for different loading conditions.

The Von Mises stress value and the contour plots of the cage under different physiological loading conditions are shown in Figure 9. The cage stress on the TSS model manifested around 130 MPa for flexion, extension, right bending, and left rotation. The TSS model illustrated a maximum stress of 166 MPa for the left lateral bending and right rotation. The APS and BPSS models showed similar stresses for axial rotation. The highest cage stress determined for the APS model was 151 MPa for extension, 125 MPa for flexion, 121 MPa for left bending. 139 MPa for right bending, 118 MPa for left rotation and 141 MPa for right rotation. The BPSS model showed 100 MPa for flexion and extension and 117 MPa for axial rotation. Although the TSS model exhibits a slightly higher stress compared to the APS or BPSS models, the difference was deemed negligible. Overall, no notable differences were detected within the Von Mises stresses on the cage for the various loading conditions.

### Stress on internal fixation

**FIGURE 10:**
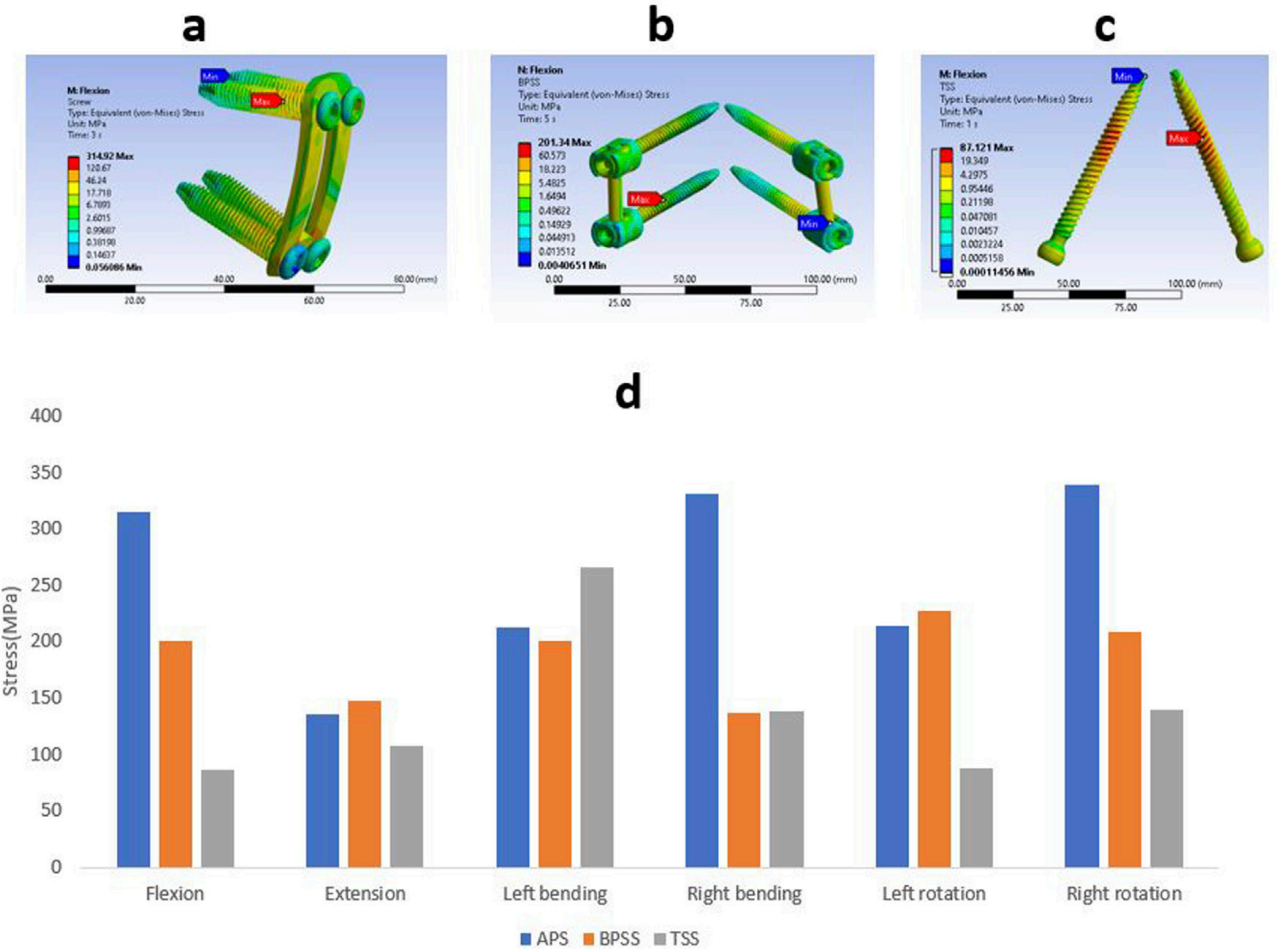
Von Mises stress contour on internal fixation under flexion condition **(A)** APS **(B)** BPSS **(C)** TSS model; **(D)** Comparison of von Mises on internal fixations for APS, BPSS, and TSS for different loading conditions.

The Von misses stress for the different internal fixation techniques under different loading conditions is shown in Figure 10. The highest stress(339 MPa) was found in right rotation for the APS model, whereas the lowest stress(87 MPa) was detected in the flexion for the TSS model. The TSS model showed lower stresses than the APS and BPSS model in all loading conditions except left bending. The left bending of the TSS model depicted a stress of 266 MPa that was 30% higher than the APS or BPSS model. The left rotation of the APS and BPSS models illustrated two-fold higher stresses than the TSS model. In extension, the stress in APS or BPSS was 30% higher than the TSS model. The stresses of the internal fixation for the APS, BPSS, and TSS models were 315, 201 and 87 MPa respectively under flexion. The highest stress for the APS model was 331 MPa for right bending, 227 MPa for left rotation in BPSS model, and 266 MPa for left bending for the TSS model. Overall, the mean stress of the TSS model manifested lower stresses on the implants compared to BPSS and APS.

### Resistance to shear load

The APS model exhibited the highest displacement of 0.86 mm, and the TSS model had the lowest displacement of 0.17 mm, whereas the BPSS model showed 0.31 mm for the same loading and boundary conditions. It was obvious that TSS models manifested better shear load resistance compared with BPSS and APS models.

## Discussion

In the current study, we established an FE model L1-sacrum, derived from a CT scan and constructed a high-grade spondylolisthesis model. The FE model was validated by comparing its ROM with other FE analysis and *in vitro* studies (Dreischarf et al., 2014; Panjabi et al., 1994). After validation, three surgical models in APS, BPSS, and TSS were created and simulated by FE software. The aim of this study is to explore the biomechanical evaluation of high-grade spondylolisthesis by focusing on parameters such as ROM, stress on adjacent annulus, cage and internal fixation stress. To the authors’ understanding, this investigation is the first to assess both anterior and posterior stabilization approaches for high-grade spondylolisthesis. In the posterior stabilization technique, a novel Transdiscal Screw was applied between L5-S1. This is also the first research to evaluate the vertebral displacement after applying shear load to these three techniques.

ROM is an essential parameter for understanding how different pathological conditions or surgical interventions affect the flexibility of the spinal functional unit. In the current study, following the implementation of internal fixation and interbody cages, the ROM of all three models was substantially decreased compared to the SP model at the fused level. The ROM of the treatment models for the L4-L5 segments increased for all loading scenarios. The SP model showed good harmony with the vitro study and healthy model except for the flexion situation. The ROM of the TSS model for the L5-S1 segment demonstrated lower than the BPSS or APS models for the flexion and extension. This information suggests proper implant selection and surgical techniques can dramatically affect the mechanical stability and function of the spine. The lesser ROM suggests that there will be less movement after the surgery as the segment will become stiffer. As the TSS model has less ROM in the fusion level, the chance of successful fusion is higher.

Another important biomechanical parameter is translational or shear resistance. Unfortunately, most biomechanical studies have totally omitted this parameter from investigation. Yet in respect to spondylolisthesis, it is probably the most important factor. The shear force is the main driving mechanical force in spondylolisthesis. Consequently, the ability to halt the slippage progression is probably the most important feature. On that respect, TSS is far more superior to APS and BPSS. We hypothesize that solid nature of TSS screws and almost orthogonal orientation to shear force are the main contributing factors. Unsurprisingly, assembled and parallelly oriented BPSS and APS constructs are relatively weak when it comes to translational resistance. We assume that higher flexional-extensional stiffness of TSS combined with better shearing resistance, substantially increases overall stiffness and leads to better fusion.

Understanding the von Mises stress distribution within the adjacent annulus is crucial for several reasons, such as analyzing degenerative effects, biomechanical stability, implant design, and clinical outcome predictions. Quantifying stress distribution within the adjacent annulus will allow clinicians to predict better which patients require specific interventions and to optimize the treatment accordingly. Excessive stress within the annulus may suggest the damage of the annulus and the risk of segmental instability, which lead to accelerated degeneration. Excessive intradiscal pressure can also lead to the disc bulging, abnormal biomechanics and ROM substantially altered. Due to altered biomechanics, can cause disc degeneration to progress to neighboring levels (Volz et al., 2022). To the authors’ knowledge, this investigation is the first to execute FEA to detect the adjacent annulus stress for anterior or posterior fixation with the interbody cage. In this present investigation, the average adjacent stress on the TSS model was relatively higher than the APS or BPSS models for all conditions. In flexion situation, the stress on the L4-L5 segment demonstrates 4.36 MPa for the TSS model, while for the APS and TSS models, it was 0.75 and 1.16 MPa, respectively. Similar stress values were also noticed for the extension scenario. Left and right bending also illustrated 100% greater stresses on the L4-L5 level in the TSS model compared with the APS and BPSS models. Interpreting this data requires caution. It may look like TSS technique puts adjacent levels (especially L4-L5) at risk. However, one should remember that there is a direct relationship between the L5-S1 stiffness and stress on the adjacent levels. Since TSS provides higher stiffness, higher adjacent level stress is expected. These data describe mechanical properties immediately after the surgery. However, the goal is to develop fusion which will eventually eliminate motion at L5-S1. Hence, there will be no difference in the adjacent annulus stress between all three models, as long as they end up with fusion.

TSS model demonstrated elevated cage stress compared with the APS or BPSS. However, it is not clear how increased stress may affect clinical outcomes. Intuitively, one may suggest that higher stress may lead to increased chances of subsidence (Dhar et al., 2023). Cage subsidence is an important factor as it is often associated with poor clinical outcomes. Biomechanical FE studies suggested that higher stress may lead cage subsidence (Chen et al., 2021). Yet this hypothesis has not been proven and considering technical difficulties may not be proven anytime soon. On the other hand, higher stress on cages may be beneficial as it may enhance bone-implant interaction and lead to better osteointegration. Another important factor that should be remembered is the size of the cage. ALIF cages used in APS and BPSS models have higher footprint areas than TLIF cages used in TSS model. Thus, under the same load one should expect higher stress in a small cage.

In this current investigation, the mean stress of the TSS screws was lower than the BPSS and APS. This phenomenon is due to reciprocal relationship between screw and cage load. TSS construct shifts the majority of load to the cage. Unloading may be beneficial since screw loosening, pullout and breakage are undesirable postoperative events. One should also remember that TSS screws are solid while APS and BPSS are assembled. Thus, the ability to withstand multiaxial dynamic load is better with TSS. Lower stress combined with solid nature of TSS puts it at significant advantage over APS and BPSS. Based on higher bone mineral density values in the posterior vertebral regions indicate that posterior surgical approaches may provide better fixation quality in elderly patients (Garay et al., 2022). As a result, TSS may be a preferable surgical option over APS.

### Limitations

This current investigation relies on FE analysis and has several limitations. Initially, the spondylolisthesis model was constructed in 3D, utilizing the CT data based on a typical lumbar spine model. In the existing model, ligaments are represented as non-linear spring elements solely influenced by tension, and muscles are not simulated. This may impact the motion and stress alterations of the lumbar spine. Secondly, a compressive force of 500N and a torque of 7.5 Nm cannot accurately replicate the cage and internal fixation stress in the patients after spinal fusion surgery. In this study, the bone mineral density was considered unique for all vertebral level. But Garay et al. (Garay et al., 2022) suggested that the bone mineral density heterogeneously distributed across regions. Hence, the bone mineral density of cancellous bone can influence the vertebra’s mechanical properties (Al-Barghouthi et al., 2020). Finally, we exclusively utilized the skeleton data from a single individual FEA simulation and did not consider variation among individuals.

## Conclusion

Overall, all three constructs demonstrated comparable biomechanical profiles. However, the solid nature of TSS, combined with better flexion-extension stiffness and high resistance to translation may be beneficial for high-grade L5-S1 spondylolisthesis. The increased stress on adjacent level with TSS model may be due to better immediate L5-S1 stabilization. As fusion develops all three models will probably end up with the same impact on adjacent levels regardless of technique.

## Data Availability

The raw data supporting the conclusions of this article will be made available by the authors, without undue reservation.
